# Multiple iliopsoas tendons: a cadaveric study and treatment implications for internal snapping hip syndrome

**DOI:** 10.1007/s00402-021-04009-5

**Published:** 2021-08-04

**Authors:** Benjamin Lin, Jonathan Bartlett, Thomas D. Lloyd, Dimitris Challoumas, Cecilia Brassett, Vikas Khanduja

**Affiliations:** 1 School of Clinical Medicine, University of Cambridge, Cambridge, UK; 2 Department of Physiology, Development and Neuroscience, University of Cambridge, Cambridge, UK; 3 Young Adult Hip Service, Department of Trauma and Orthopaedics, Addenbrooke’s - Cambridge University Hospital, Cambridge, CB2 0QQ UK

**Keywords:** Internal snapping hip syndrome, Iliopsoas, Anatomical variation, Hip arthroscopy, Iliopsoas tenotomy

## Abstract

**Purpose:**

This cadaveric study aimed at describing the anatomical variations of the iliopsoas complex.

**Methods:**

The iliopsoas complex was dissected unilaterally in 28 formalin-embalmed cadavers—13 males and 15 females with a mean age of 85.6 years. The number, courses and widths of the iliacus and psoas major tendons were determined. Patients with previous hip surgery were excluded. The following measurements were taken from the mid-inguinal point: the distance to the point of union of the psoas major and iliacus tendon; and the distance to the most distal insertion of iliopsoas.

**Results:**

The presence of single, double and triple tendon insertions of iliopsoas were found in 12, 12 and 4 of the 28 specimens, respectively. When present, double and triple tendons inserted separately onto the lesser trochanter. The average length of the iliopsoas tendon from the mid-inguinal point to the most distal attachment at the lesser trochanter was 122.3 ± 13.0 mm. The iliacus muscle bulk merged with psoas major at an average distance of 24.9 ± 17.9 mm proximal to the mid-inguinal point. In all cases, the lateral-most fibres of iliacus yielded a non-tendinous, muscular insertion on to the anterior surface of the lesser trochanter and the femoral shaft, rather than joining onto the main iliopsoas tendon(s). The average total width of the psoas major tendon decreased with an increasing number of tendons: 14.6 ± 2.2 mm (single tendon), 8.2 ± 3.0 mm (2 tendons present) and 5.9 ± 1.1 mm (3 tendons present) (*P* < 0.001).

**Conclusions:**

The results of this study suggest that multiple tendinous insertions of iliopsoas are present as an anatomical variant in more than 50% of the population. The non-tendinous muscular insertion of the iliopsoas on to the anterior surface of the lesser trochanter and femoral shaft found represents a novel anatomical variant not previously described.

**Level of evidence:**

Level V

## Introduction

In recent years, the iliopsoas muscle complex has gained increasing recognition as a generator of hip pain in internal snapping hip syndrome (ISHS) [1]. This syndrome describes the painful and audible movement of the iliopsoas tendons across the hip joint, with the snapping thought to arise from movement over the iliopectineal eminence or the femoral head. Though the exact aetiology of this condition is not known, it has been speculated that anterior instability of the femoral head in the acetabulum with characteristic 3 o’clock anterior labral injury leads to tensioning of iliopsoas with resultant pain and snapping [2–4].

Although initial management of ISHS is conservative—consisting of analgesia, physiotherapy and life-style modification—tenotomy may be indicated in cases of persistent debilitating pain [5, 6]. This procedure, however, is only considered in the most severe cases due to previous findings of post-operative hip flexion weakness, atrophy of the remanent iliopsoas, and concerns regarding gross instability of the hip joint following iliopsoas tenotomy [5, 7–9]. Tenotomy can be performed at the level of the lesser trochanter or at the level of the labrum, and as an open procedure or arthroscopically, with better outcomes documented for the latter [5, 10]. While this may in part be due to the concomitant treatment of intra-articular pathologies, arthroscopic tenotomy also shows reduced failure rates, complications and post-operative pain.

Despite improvements in the treatment of ISHS, post-tenotomy recurrence has been well-documented [11]. It is thought that this may be due to the presence of multiple tendons, in that only one of the pathological tendons would be released by the tenotomy [1, 12]. Though this hypothesis is supported by the increasing recognition of the prevalence of multiple tendons of iliopsoas, there have been no detailed studies of the type or frequency of this variation in whole body cadavers [11–13].

Previous studies have highlighted the anatomical complexity and variability of the iliopsoas tendon, thus calling into question the traditional view of the iliopsoas tendon as a single conjoint tendon formed by the fusion of iliacus and psoas major which inserts onto the lesser trochanter [12, 14]. Thus, a more detailed description of the anatomy of iliopsoas, its tendons and their insertions is of clinical significance in view of the causative role of this tendon complex in ISHS. The purpose of this cadaveric study was, therefore, to describe the anatomical variations of the iliopsoas complex. We hypothesized that multiple tendons of iliopsoas are more common than single tendinous insertion.

## Methods

A total of 28 formalin-embalmed cadavers (13 males and 15 females, with no lower limb abnormalities or a history of hip surgery), with a mean age of 85.6 years (range 70–99), were dissected. Informed consent had been obtained from the donors before decease for the use of their bodies for anatomical education, training and research under the Human Tissue Act 2004. Dissections were performed unilaterally (13 left, 15 right) as the contralateral limb had already been dissected for undergraduate teaching. Patients with previous hip surgery were excluded from the study. The joint capsule was not investigated. All dissections were performed under the supervision of two anatomy demonstrators (TL and DC) by the two primary authors (JB and BL) who were medical students at the time of this study.

**Fig. 1:**
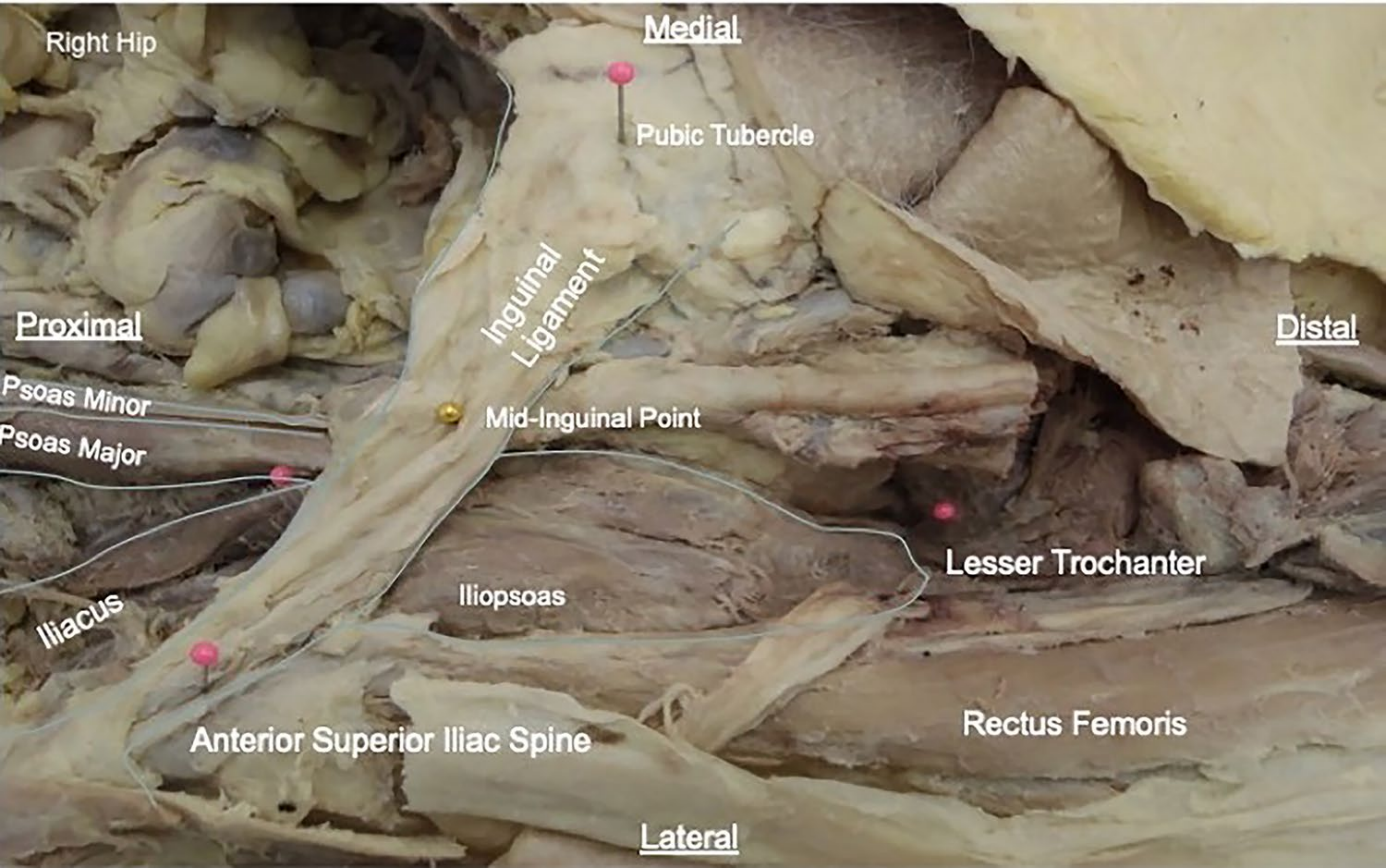
Example of iliopsoas dissection. A labelled example dissection of the left iliopsoas complex from the abdomen (proximal, left) through to the insertion of the lesser trochanter (distal, right). Viewed from anterior aspect in coronal plane

Midline abdominal and bilateral subcostal incisions were made, with removal of the abdominal viscera to allow visualisation of the iliopsoas muscle on the posterior abdominal wall. The origin of psoas major was visualised. The iliopsoas muscle was then dissected in its entirety from its proximal origin within the abdominal cavity to its distal insertion at the lesser trochanter and anterior femoral shaft. Proximally, this involved removal of the retroperitoneal fascia to visualise the separate bodies of iliacus and psoas major. Distally, the skin was removed with the inguinal ligament preserved superiorly (Fig. 1).

The origins of rectus femoris and sartorius were incised and their muscle bulks reflected medially. Tensor fasciae latae was partially resected at its origin, allowing lateral reflection and clear visualisation of the iliopsoas muscle complex. To improve visualisation of the insertion at the lesser trochanter, the neurovascular bundle within the femoral triangle and the medial compartment of the thigh were also removed. Following dissection, the number of iliacus and psoas major bodies were documented. The presence, course and insertion of psoas minor were also noted.

**Fig. 2:**
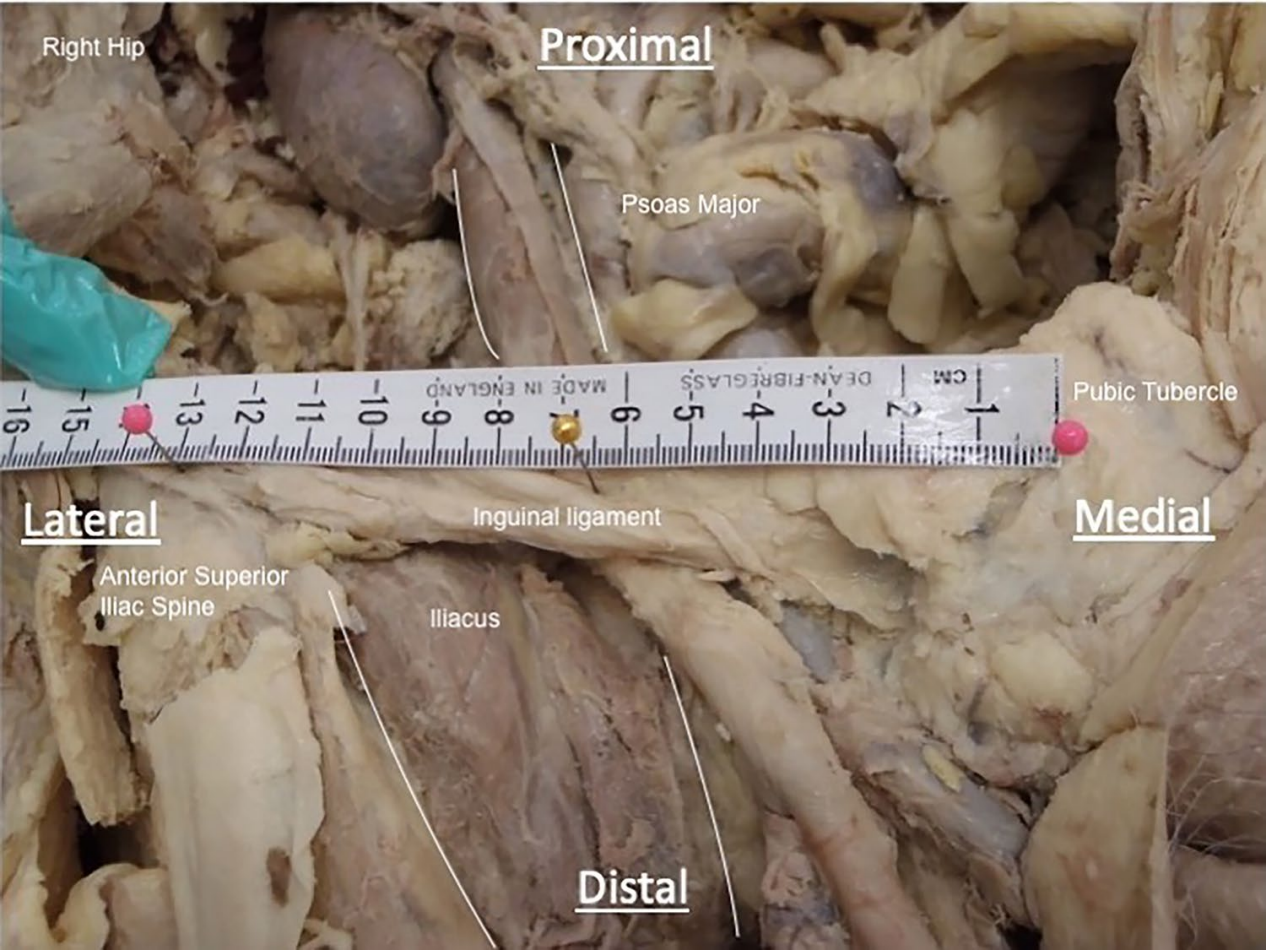
Example of mid-inguinal point dissection. The location of the right mid-inguinal point (central pin) measured halfway between the anterior superior iliac spine (left pin) and the pubic tubercle (right pin) along the preserved inguinal ligament. Viewed from anterior aspect in coronal plane, perpendicular to body axis

The mid-inguinal point was chosen as a reproducible, easily located and identifiable landmark. This was located using a tape measure and marked with a pin (Fig. 2), and the following were measured using Fuller 150 mm Digital Vernier Calipers (Fuller, Quebec, Canada):

-The distance between the mid-inguinal point and the point of union of the muscle bodies of psoas major and iliacus
-The distance between the mid-inguinal point and the most distal insertion of iliopsoas on the lesser trochanter

**Fig. 3:**
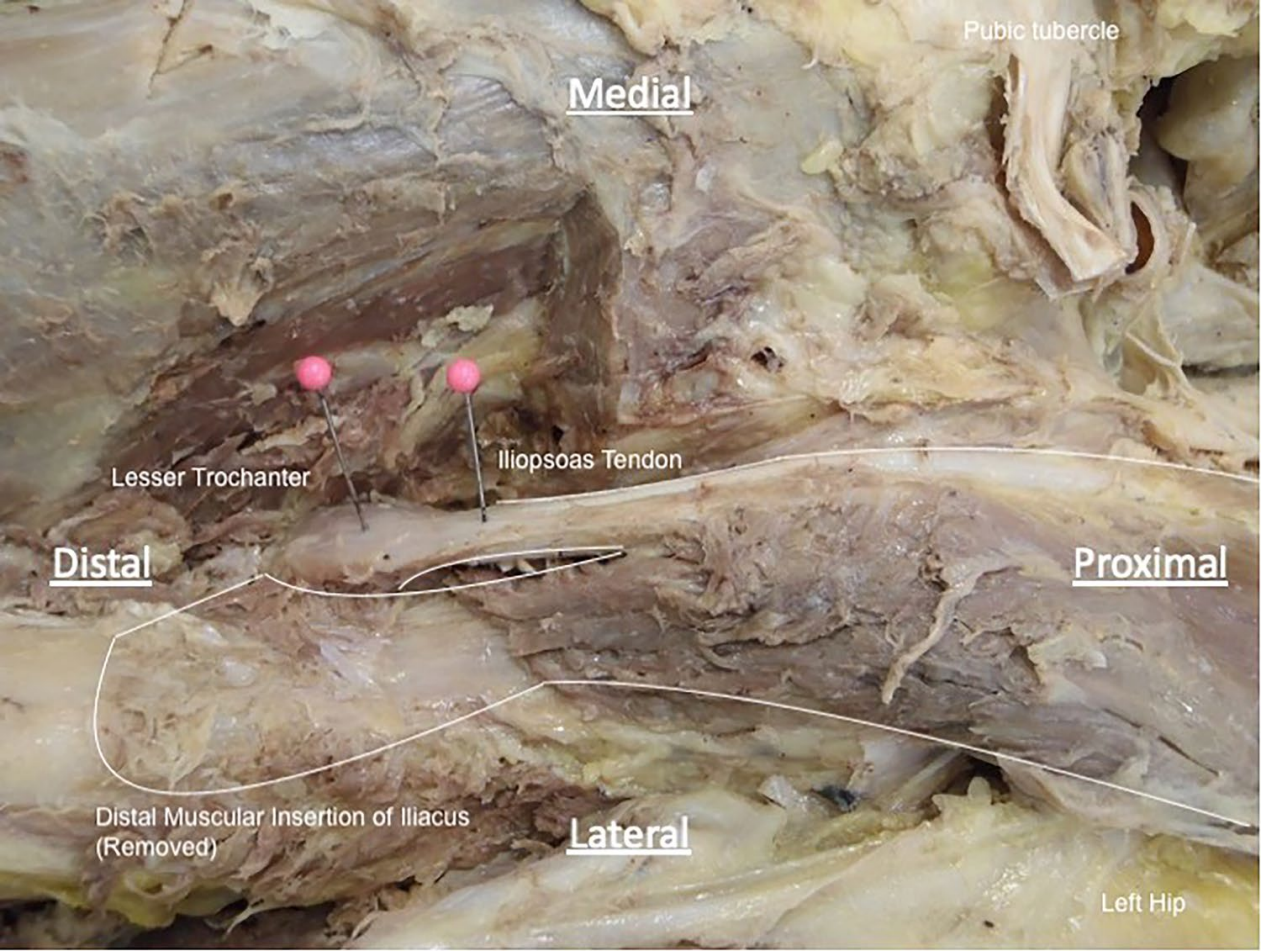
Example of point at which iliopsoas tendons were cut. A dissection of the left iliopsoas complex to demonstrate the point at which the iliopsoas tendon was cut (represented by the pin on the right). This was 2 cm proximal to the distal-most insertion of the iliopsoas complex. The location of the non-tendinous, muscular insertion has been highlighted—this was removed in this specimen to enable measurement of the iliopsoas tendon, though the remanent muscle fibres can be seen. Viewed from anterior aspect in coronal plane, perpendicular to body axis

A point 2 cm proximal along iliopsoas from its most distal insertion on the lesser trochanter was identified using the digital calipers and marked with a pin. This was selected as the most easily reproducible point to account for the fanning out of the tendon at its insertion (Fig. 3). The muscles were then cut at this point and lifted to more accurately characterise the number of tendons present and to track them back to their original muscle bulk. The widths of the tendons were measured in the widest plane at this point.

All data analysis was performed in FigurePad™ (Prism 7; Graphpad Software Inc., La Jolla, CA, USA). As a normal distribution could not be assumed, all comparisons were performed using Kruskal–Wallis with Dunn’s Multiple Comparison Tests. A *P* value of < 0.05 was chosen as significant. All measurements were taken 3 times, under observation by a colleague, and an average value of the three measurements used. All data are presented as mean ± standard deviation.

## Results

### Number and origin of tendinous insertions

**Fig. 4:**
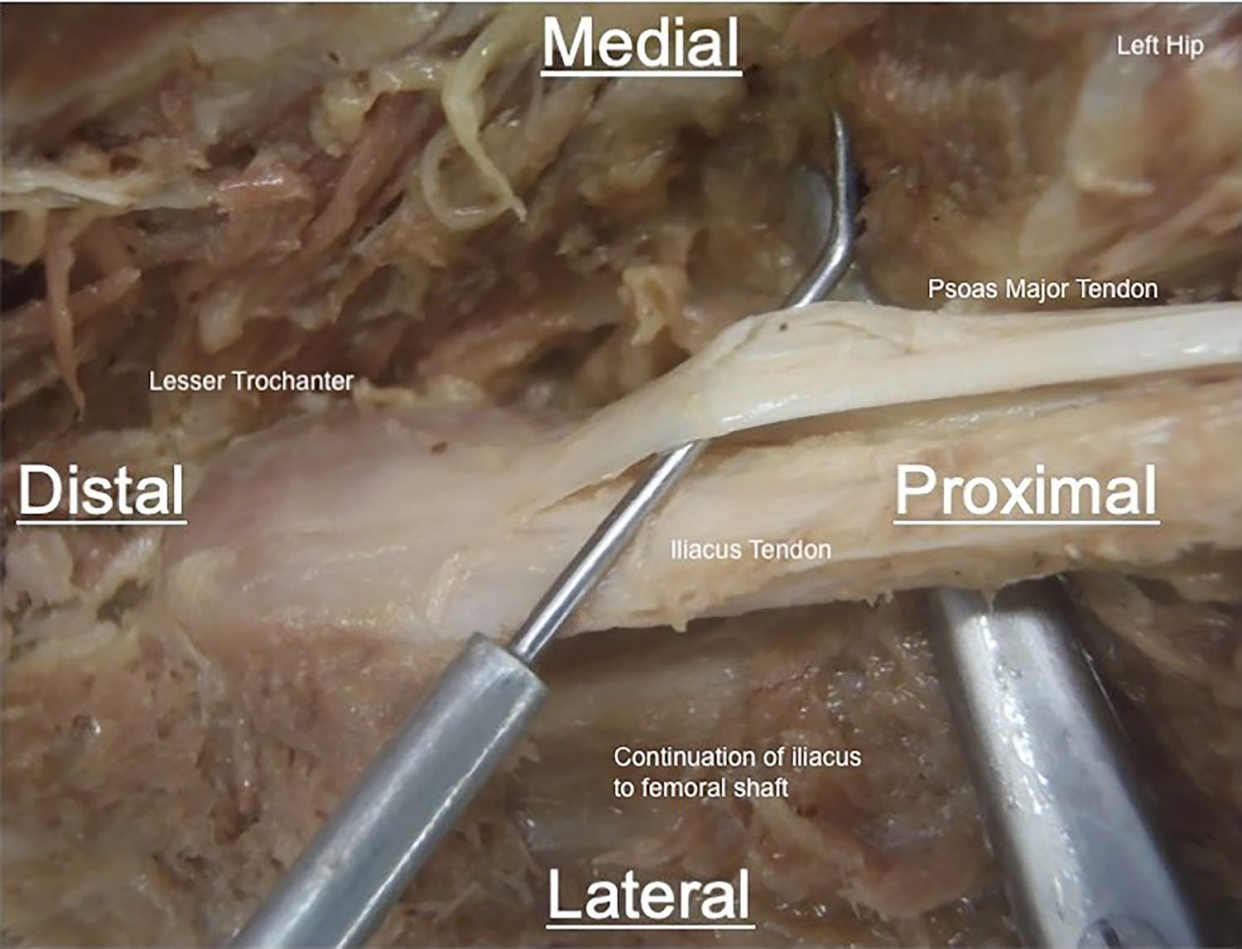
Example of dissection of a double iliopsoas tendon variant. A dissection of the left iliopsoas demonstrating a double tendinous insertion of iliopsoas onto the lesser trochanter. Viewed from anterior aspect in coronal plane, perpendicular to body axis

**Fig. 5:**
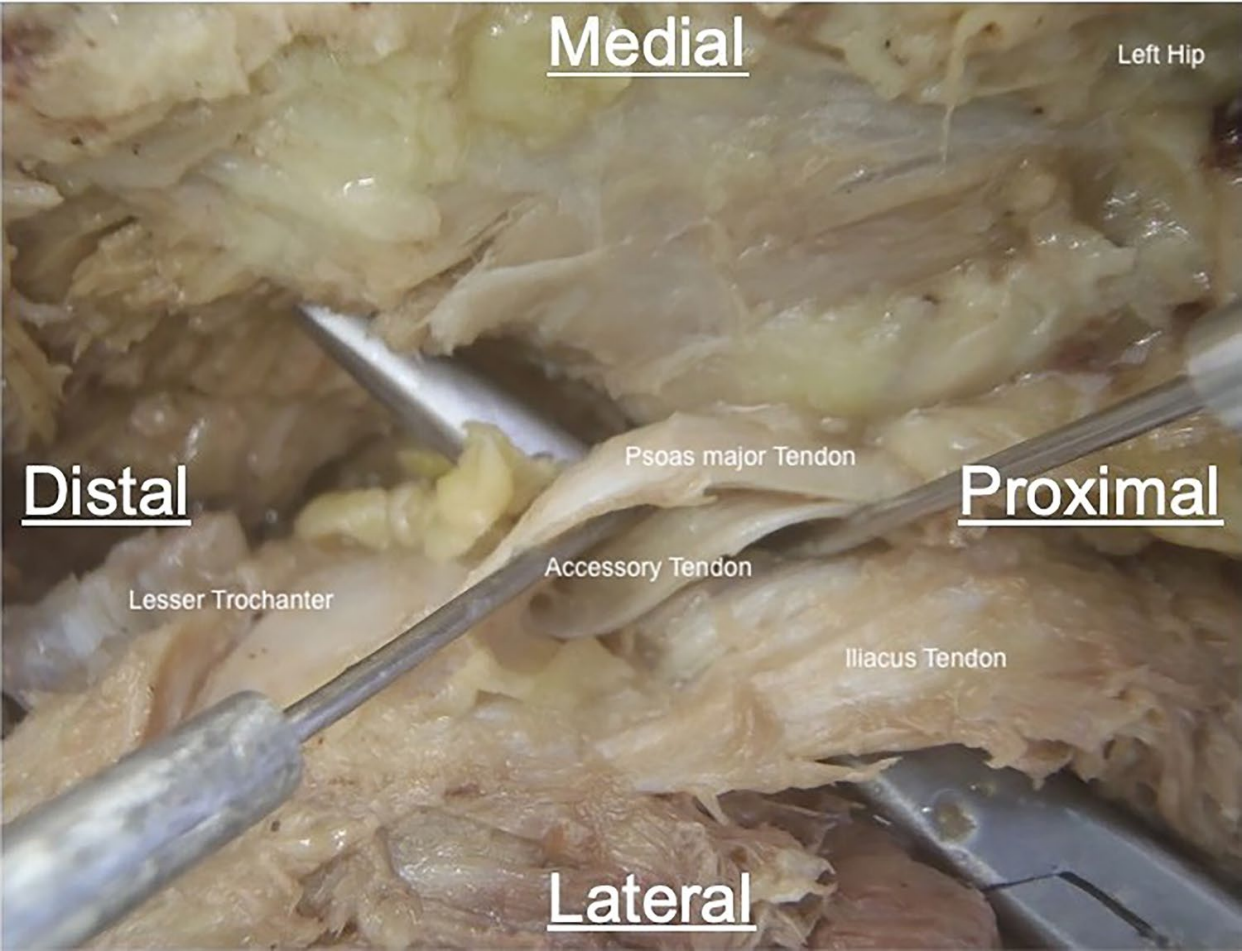
Example of dissection of a triple iliopsoas tendon variant. A dissection of the left iliopsoas demonstrating a triple tendinous insertion of iliopsoas onto the lesser trochanter. The medial most tendon originated from the body of psoas, whilst the remaining two originated from iliacus. Viewed from anterior aspect in coronal plane, perpendicular to body axis

Of 28 cadavers, single, double and triple tendinous insertions of iliopsoas onto the lesser trochanter were found in 12 (43%), 12 (43%). and 4 (14%) subjects, respectively (Figs. 4 and 5). This insertion was found to be at the most anteromedial point of the lesser trochanter. There was no evidence of damage or degeneration of the iliopsoas in any specimen. When double or triple tendons were found, these inserted separately onto the lesser trochanter.

In all specimens with a single tendinous insertion, the tendon was found to arise from the psoas major muscle bulk with iliacus merging with the psoas muscle proximally (Table 1). Additionally, in all instances where more than one tendon inserted onto the lesser trochanter, the medial tendon arose from psoas major and the lateral one from iliacus. In cadavers with three tendons, the medial tendon arose from psoas major, with the other two originating from iliacus.

**Table 1:** Measured distance from the mid inguinal point

	Distance from mid inguinal point (mm)^a^
Tendon length to the lesser trochanter	122.3 ± 13.0
Point of fusion of iliacus and psoas major	− 24.9 ± 17.9

^a^Positive values indicate a point distal to mid inguinal point and negative values indicate a point proximal to mid inguinal point

### Width of tendons

The average total width of the psoas major tendon decreased with an increasing number of tendons (Table 2). The tendon of psoas major was found to be significantly smaller if there were multiple tendons present. Psoas major tendons were significantly smaller than iliacus tendons.

**Table 2:** Width of iliacus, psoas major and accessory tendons

	Single tendon	Double tendon	Triple tendon
Cumulative tendon (mm)	14.6 ± 2.2	23.4 ± 4.3^a^	21.9 ± 4.0^a^
Psoas major tendon (mm)	14.6 ± 2.2	8.2 ± 3.0^a^	5.9 ± 1.1^a,b^
Iliacus tendon (mm)	−	15.2 ± 4.2^c^	9.2 ± 3.7^b,c^
Accessory tendon (mm)	−	−	6.8 ± 3.0

^a^*P* < 0.001 when compared with single tendon widths

^b^*P* < 0.001 when compared with double tendon widths

^c^*P* < 0.001 when compared with psoas major widths from same group

### Psoas minor

Psoas minor was present in 50% of cadavers and did not follow the iliopsoas muscle complex distal to the inguinal ligament. In all such cases, the psoas minor tendons inserted into the fascia overlying the iliacus muscle bulk, the inguinal ligament and the iliopubic eminence.

### Iliacus

**Fig. 6:**
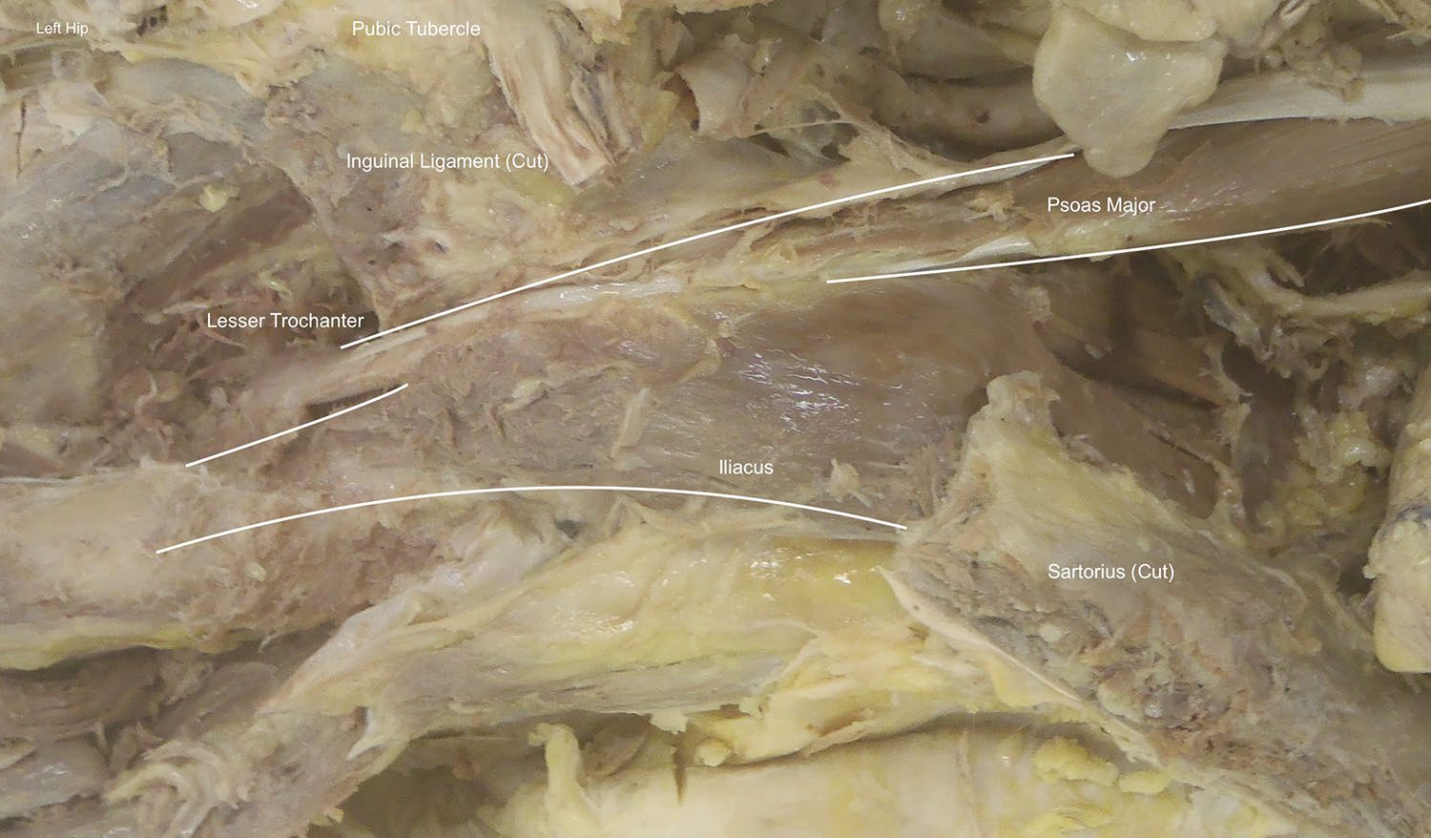
Example of dissection of accessory anterior insertion of iliacus. A dissection of the left hip to demonstrate the non-tendinous muscular insertion of iliacus onto the femoral shaft (highlighted). Some of the muscle fibres have been removed to allow access to the iliopsoas tendons. Viewed from anterior aspect in coronal plane, perpendicular to body axis

In all cases, iliacus arose from the whole of the iliac fossa, tracking down beneath the inguinal ligament to insert on the anteromedial surface of the lesser trochanter. In all cases, the majority of fibres from iliacus merged with psoas major, but the lateral-most fibres yielded a non-tendinous, muscular insertion on to the anterior surface of the lesser trochanter and in the infra-trochanteric region of the femoral shaft (Fig. 6). Furthermore, iliacus was found to be composed of two distinct muscle bodies, separated by fascia in 15 (53.6%) specimens. In the specimens where dual muscle bulks were found, the medial muscle bulk was found to partially overlie the lateral bulk and was located posterior to psoas major in all cases.

## Discussion

The most important finding of the present study was the high prevalence of multiple iliopsoas tendons. Of the 28 cadavers dissected in this study, over half were found to have multiple iliopsoas tendons, with two tendons in 12 subjects (43%) and three tendons in 4 (14%) subjects. This is the largest study to utilise whole body cadavers to analyse the path and variation of the iliopsoas complex. These findings support the growing evidence that multiple tendons are not a rare anatomical variant. These results confirm our hypothesis that the iliopsoas tendon is more commonly composed of more than one tendon than a single tendinous insertion. Furthermore, we were able to demonstrate a high prevalence of anatomical variations of iliacus, psoas major and psoas minor.

Previous studies using hemipelves and imaging have demonstrated a similar prevalence of multiple tendons to that found in this study [12, 13]. Philippon et al. found multiple tendons to be present in over 70% of sectioned frozen hemipelves, and that in 7.5% of cadavers, three tendons could be found [12]. In a study of 10 fresh frozen cadavers, Gómez-Hoyos et al. [14] reported 7 cases with two tendinous insertions. They did not, however, report any triple tendon cases. As in our study, the authors noted the iliopsoas complex insertion to be at the most anteromedial aspect of the lesser trochanter. Using data from magnetic resonance arthrograms, Polster et al. noted the appearance of separate tendons within the iliacus muscle in 14 out of 12 patients [13]. To further assess this finding, these authors performed three cadaveric dissections, finding an additional tendon in one of these cadavers. Unfortunately, due to the small number of dissections performed, the authors were unable to further speculate regarding the prevalence of multiple iliopsoas tendons. A further study assessed the presence of multiple iliopsoas tendons in 28 patients undergoing endoscopic trans-capsular release of iliospsoas, finding 4 patients with bifid tendons and one patient with a triple tendon variant [11]. None of these were documented on pre-operative magnetic resonance arthrograms and only 60% were found on retrospective analysis, thus highlighting the limitations of imaging when assessing the morphology of the iliopsoas tendon complex.

In keeping with previous studies, we found significant differences in the width of individual iliopsoas tendons depending on the number of tendons present, as well as differences in the width of the total tendon complex [12]. Although Philippon et al. suggested that a second tendon should be sought if the initial tendon encountered measures less than 1 cm, given the high prevalence of double tendons, we would recommend that a second tendon should always be sought [12]. Furthermore, arthroscopic surgeons should be aware of the possibility of three iliopsoas tendons as these are by no means infrequent (14.3% in this study). Care must be taken when searching for additional tendons, however, due to the proximity of the medial femoral circumflex artery to the medial edge of the psoas tendon sheath [6, 15].

Traditionally, iliopsoas has been considered to be formed by the progressive fusion of the single muscle bulks of iliacus and psoas majors into a single tendinous insertion [13]. In our study, we found that in half of our subjects, two iliacus bodies could be found. Additionally, a distinct psoas minor tendon was present in half of the subjects. When present, psoas minor inserted into the pelvic bowl and did not follow the course of psoas major. Additionally, iliacus was found to have a component inserting onto the anterior portion of the proximal femoral shaft in all specimens, a finding absent from previous cadaveric studies.

The role of multiple iliopsoas tendon in ISHS remains an area of debate [16, 17]. A study using dynamic sonography in 18 snapping hips suggested that this pathology could be due to the trapping of the iliopsoas tendon on the anterior surface of the iliacus muscle body and its abrupt collision with the pubic bone upon lateral release [1, 4, 6]. Additionally, they suggest that in a subset of patients, the snapping is the result of two separate iliacus tendon heads abruptly abutting one another. This mechanism has since been contested in a case report which argued that removal of a single tendon should cause resolution of the snapping symptoms and highlighted a patient with a bifid tendon whose symptoms did not resolve following a single tenotomy, but did so following a revision tenotomy [18]. Though Philippon et al. suggests that post-tenotomy recurrence of ISHS in patients with bifid tendons could be explained by the presence of triple tendons, drawing general conclusions from a single case study is unwise and the initial mechanism of ISHS in this patient may not have been the result of bifid tendon abutment [12].

The most effective location for iliopsoas tenotomy remains uncertain, with particular concerns noted regarding the risk of post-operative weakness. Previous MRI studies have demonstrated both higher rates and greater degrees of iliacus atrophy when tenotomy is performed at the level of the lesser trochanter when compared to the labrum [7, 16, 19]. These studies also reported a 25% rate of gluteus maximus atrophy and 35% rate of chronic iliopsoas tendon disruption in post-operative MRI when the tenotomy was performed at the lesser trochanter.

Neither of these findings were demonstrated in those who underwent tenotomy at the level of the labrum. Another study, however, has demonstrated almost 20% decrease in hip flexion strength and a 25% decrease in iliopsoas muscle strength when performing a tenotomy at the level of the labrum [5]. A further study found this weakness to have improved by 8 weeks post-operatively [11].

There are also additional risks associated with each approach. Iliopsoas tenotomy at the level of the labrum necessitates a large medial interportal capsulotomy, making repair both a technical challenge and prone to failure [20]. This also increases the likelihood of capsulolabral and capsule-iliopsoas scarring. For tenotomy at the lesser trochanter, either an anterior approach or posterior trans-quadratus femoris approach can be utilised. The former is associated with inherent risk to the femoral nerve and lateral femoral cutaneous nerve, and the latter with an inherent risk to the medial femoral circumflex artery or the first perforator branch of profunda femoris [21]. Furthermore, gross instability of the hip has been documented post-operatively [8, 9].

The strength of this study includes the use of a moderate number of whole cadavers and a more extensive dissection than in previous studies. This allowed us to visualise the whole course of the iliopsoas muscle complex and prevented distortion of the anatomy during the production of hemi-pelves.

### Limitations

There are, however, several limitations to this study. First, the age of the cadavers used was significantly higher than patients undergoing iliopsoas tenotomy. This will likely have an impact on the size of the iliopsoas tendons. Furthermore, we had no indication as the whether the cadavers used in this study had a history of internal snapping of the hip. As such, anatomical difference between our cohort and patients with symptomatic snapping undergoing arthroscopy may be different. We were unable to interrogate the iliopsoas tendon complex on the contralateral side of the cadavers as this had already been dissected for undergraduate teaching. This means that we were unable to assess if multiple tendon variants are a bilateral or unilateral phenomenon.

In order to ensure measurement were reproducible the inguinal ligament and a point 2 cm from the distal insertion were used as landmarks for measurements. These, however, do not represent landmarks visualised arthroscopically and their ability to be used for intraoperative comparison is therefore limited. Furthermore, though all measurement were taken by two authors there remains a risk of interobserver variability when measuring this landmark. This variability is also present when measuring the point of fusion of iliacus and psoas major as this point was not always clearly defined.

Furthermore, several factors may have altered the dimension of iliopsoas when compared to its size in vivo. The formalin used in the embalming process may have altered the dimensions of the muscular components of iliopsoas surrounding it. The embalming process also meant we were unable to fully examine the hip joint with regards to range of motion, the capsule or the role of pelvic positioning on the position and course of iliopsoas. However, these alterations are unlikely to have affected the presence of multiple tendons due to the tendon sheaths found to envelop them. As the remainder of the body needed to be preserved for undergraduate teaching, we were unable to perform further dissection to assess the pelvic incidence, pelvis tilt, sacral slope, femoral version, acetabular version, neck-shaft angle or lumbar spine malalignment. These factors may have influenced the position and course of iliopsoas.

## Conclusions

The results of this study suggest that multiple tendinous insertions of iliopsoas are present as an anatomical variant in more than 50% of the population. The non-tendinous muscular insertion of the iliopsoas on to the anterior surface of the lesser trochanter and femoral shaft found represents a novel anatomical variant not previously described.
